# CRISPR/Cas9技术在血液肿瘤CAR-T细胞疗法中应用策略的研究进展

**DOI:** 10.3760/cma.j.cn121090-20240911-00343

**Published:** 2025-05

**Authors:** 郁文 王, 永民 汤

**Affiliations:** 浙江大学医学院附属儿童医院血液肿瘤中心，浙江省儿童白血病诊治技术研究中心，国家儿童健康与疾病临床医学研究中心，杭州 310003 Department/Center of Hematology-oncology, Children's Hospital of Zhejiang University School of Medicine; Pediatric Leukemia Diagnostic and Therapeutic Technology Research Center of Zhejiang Province; National Clinical Research Center for Child Health, Hangzhou 310003, China

## Abstract

嵌合抗原受体T细胞（CAR-T细胞）疗法在复发难治性B细胞恶性肿瘤治疗中取得了突破性进展，但仍面临制备复杂、适应证局限、细胞耗竭及疗效不持久等挑战。CRISPR/Cas9作为一种高效、相对简单的基因编辑技术，为突破这些瓶颈提供了新的可能。本文简述CRISPR/Cas9的工作机制，重点探讨其在开发通用型CAR-T细胞、增强T细胞功能及拓展CAR-T细胞至T系和髓系白血病治疗中的最新应用与临床实践。此外，本文聚焦近两年用于提高CRISPR/Cas9系统的编辑精准性、递送效率和安全性的优化策略，以期为CAR-T细胞疗法的优化设计和临床应用提供参考。

## Abstract (en)

Chimeric antigen receptor (CAR) T-cell therapy has achieved breakthroughs in treating relapsed/refractory B-cell malignancies. However, it still faces challenges, including complex manufacturing processes, limited indications, T-cell exhaustion, and insufficient durability of therapeutic efficacy. CRISPR/Cas9, a highly efficient and relatively simple gene-editing technology, offers new avenues for overcoming these limitations. This review briefly outlines the working mechanism of CRISPR/Cas9 and focuses on its recent applications and clinical practices in developing universal CAR T-cells, enhancing T-cell function, and extending CAR T-cell therapy to T-cell and myeloid leukemias. Furthermore, this review highlights optimization strategies developed over the past two years to enhance the editing precision, delivery efficiency, and safety of the CRISPR/Cas9 system, aiming to provide insights for the optimal design and clinical application of CAR T-cell therapy.

嵌合抗原受体（CAR）T细胞在难治性B细胞恶性肿瘤治疗中取得突破，截至2024年9月1日，美国食品药品监督管理局已批准6款治疗B细胞恶性肿瘤的CAR-T产品[1]–[6]，其中4款靶向CD19，2款靶向BCMA[6]。我国国家药品监督管理局在近两年批准了另外3款CAR-T产品[7]–[9]。围绕CAR-T细胞的研究与日俱增，但CAR-T应用效果的局限、细胞耗竭、相关严重不良反应等挑战仍待突破，CRISPR/Cas9基因编辑技术或能解决这些难题。大量研究投入到CRISPR/Cas9提升CAR-T效能以及优化CRISPR/Cas9的效率与安全性中。本文简要阐述CRISPR/Cas9的工作机制，着重介绍近年来应用CRISPR/Cas9改善CAR-T细胞治疗血液肿瘤的策略，讨论提高CRISPR/Cas9精准度与安全性的研究进展，为进一步使用基因编辑工具优化CAR-T细胞疗法提供设计与应用的考虑要点。

一、CRISPR/Cas9技术的工作机制

以目前使用最广泛的化脓性链球菌Cas9蛋白[10]为例，CRISPR/Cas9技术包含两个重要组分——Cas9蛋白与向导RNA（guide RNA, gRNA），其在真核细胞内进行基因编辑的过程可分为识别、剪切与修复三个阶段[11]。识别：细胞核内gRNA和Cas9蛋白形成的Cas9/gRNA核糖核蛋白复合物（ribonucleoprotein, RNP）在gRNA引导下与目标DNA序列匹配，gRNA∶DNA互补程度影响Cas9的核酸内切酶活性。剪切：gRNA与DNA靶向链高度互补时，Cas9的两个核酸酶结构域HNH和RuvC分别剪切DNA靶向链及其互补DNA链，造成DNA双链断裂（double-strand breaks, DSB）。修复：CRISPR/Cas9实现基因编辑主要依赖DSB诱导的细胞内源性DNA修复机制工作，包括非同源末端连接（non-homologous end joining, NHEJ）和同源定向修复（homology-directed repair, HDR）。NHEJ机制可能引起随机插入或缺失（indels），导致移码突变或引入终止密码子，使目的基因编码的蛋白失活，从而实现基因敲除；HDR以同源序列进行修复，其精确性高于NHEJ，当大量与编辑位点上下游高度同源的模板存在时，经该途径可将外源序列整合至基因组实现目的序列敲入。理论上，通过设计合理的gRNA可靶向绝大部分基因座，结合天然或经修饰的Cas9蛋白后能对目标基因进行修改或调控。

二、CRISPR/Cas9在血液肿瘤CAR-T细胞疗法中的应用

1. 通用型CAR-T：全球上市的CAR-T均为自体CAR-T细胞[12]，因多线化疗对免疫系统的打击，患者来源CAR-T细胞中与长期疗效正相关的记忆样细胞比例较低[13]，10％～20％的患者在临床试验中CAR-T细胞制备失败[14]，CAR-T制备过程意外产生“CAR-肿瘤细胞”导致患者复发死亡案例也有报道[15]。因此开发具备即用型、细胞活性好、成本较低优点的通用型CAR-T细胞是该领域的重要研究方向。

通用型CAR-T指通过基因工程改造健康供者T细胞，使其成为可供多名患者使用的同种异基因CAR-T细胞。异基因伴随的移植排斥反应［移植物抗宿主病（GVHD）和宿主抗移植物反应（host-versus-graft reaction, HVGR）］是限制通用型CAR-T发展的主要障碍。多项研究在探索利用CRISPR/Cas9技术减少通用型CAR-T对受者正常组织的损伤和（或）受者对CAR-T的免疫排斥。

通用型CAR-T产生的GVHD主要为供者T细胞的TCR识别受者HLA，导致受者正常组织损伤，因此可利用CRISPR/Cas9敲除TCR基因的保守区TRAC或TRBC来降低GVHD的发生风险。Ottaviano等[16]结合慢病毒载体与电转技术向供者来源T细胞内导入CD19-CAR与靶向TRAC和CD52的CRISPR/Cas9系统，获得TCR<1％、CD52<30％的通用型CD19 CAR-T细胞，6例复发/难治B细胞急性淋巴细胞白血病（r/r B-ALL）患儿接受回输，1例出现皮肤GVHD；4例流式细胞术评估完全缓解（CR）获得桥接造血干细胞移植（HSCT）机会，其中2例CR分别持续9个月和18个月；CAR-T在接受移植患者中留存时间无法评估，在未接受HSCT患者体内留存时间不足1个月。另一项成人Ⅰ期CD19/CD22双靶通用型TCR敲除与CAR-T临床试验中，以RNP形式导入CRISPR/Cas9系统敲除TRAC及CD52的效率相似，联合阿伦单抗预处理，该研究无GVHD发生，CR/血液学恢复不完全的CR（CRi）率为83.3％（5/6），中位随访4.3个月，3例维持微小残留病（MRD）阴性，CAR-T细胞体内中位留存时间为42 d[17]。通过CRISPR/Cas9将CAR定点整合至TRAC位点，在一步操作中同时实现TCR敲除与CAR导入所制备的通用型CD19 CAR-T细胞，在27例大B细胞淋巴瘤（LBCL）治疗中未观察到GVHD发生[18]。类似原理制备的通用型CAR-T细胞在治疗6例B细胞非霍奇金淋巴瘤的研究中也无GVHD报道[19]。

目前临床研究表明CAR-T细胞引发的GVHD基本可由基因编辑手段解决，受者TCR识别CAR-T细胞HLA导致CAR-T细胞因HVGR被清除成为更需解决的问题[20]–[21]。部分研究采用CD52单抗联合CD52敲除的CAR-T细胞，以降低宿主免疫细胞总量，减轻HVGR，同时不影响CAR-T功能，但通用型CAR-T体内留存情况仍不够理想[16]–[17],[22]。得益于CRISPR/Cas9多重编辑的优势，同时编辑CAR-T细胞的TCR与HLA可解决GVHD与HVGR，提高通用型CAR-T的适用性。HLA-Ⅰ类分子由高度多态性的α链及B2M基因编码的β_2_微球蛋白组成，HLA-Ⅱ类分子由HLA-DR、DP和DQ基因编码的α链和β链构成。通过CRISPR/Cas9敲除B2M和HLA-Ⅱα链编码基因（HLA-DRA、DQA和DPA）[23]或CIITA（MHCⅡ类反式转录激活因子）[24]以移除CAR-T细胞表面HLA-Ⅰ类和Ⅱ类分子，可明显降低CAR-T的免疫原性，且HLA-Ⅰ/Ⅱ缺失并不影响CAR-T细胞活化与细胞毒效应功能的发挥。经CRISPR/Cas9移除TCR和HLA-Ⅰ的通用型CD19 CAR-T细胞治疗r/r LBCL数据显示，接受300×10^6^个细胞以上剂量患者的总体反应率（ORR）和CR率分别为67％（18/27）和41％（11/27），近50％ CR患者维持6个月以上，其中2例24个月持续CR，试验过程无GVHD发生，该研究目前已进入Ⅱ期[18]。值得注意的是，完全缺失HLA的CAR-T因缺乏NK细胞抑制信号而可能被活化NK细胞清除，过表达siglec7配体[25]、携带异体免疫防御受体[26]、CD64[27]或CD47[24]等在荷瘤模型上表现出保护细胞不受先天免疫细胞攻击的作用，但临床效果还有待进一步探究。

2. 改善CAR-T细胞功能：在肿瘤抗原长期刺激与免疫抑制微环境等多种因素作用下，CAR-T细胞出现细胞耗竭状态，表现为CAR-T增殖及存活能力减弱、细胞毒性作用降低、分泌细胞因子能力下降，无法有效杀伤肿瘤，常伴随多种免疫抑制分子上调[28]。许多CAR-T临床研究表明CAR-T细胞耗竭与治疗失败密切相关[29]，借助CRISPR/Cas9技术对CAR-T细胞实现特定基因的敲除或敲入，改变CAR-T的基底信号（tonic signaling）、细胞代谢、分化及免疫抑制信号等来增强CAR-T细胞效能，以提高CAR-T疗法的长期效果。

CAR通常以病毒载体随机整合至基因组的方式导入T细胞，大多数CAR存在胞外段正电荷斑块介导自发聚集产生基底信号的现象，基底信号过强是CAR-T接触肿瘤前就出现耗竭的可能机制[30]。将CAR经CRISPR/Cas9定向至TRAC使CAR在内源启动子驱动下表达，让CAR在T细胞膜上表达更为均质，从而降低CAR-T细胞基底信号强度，使CAR-T在多次抗原暴露下仍能动态调节CAR的内化与再表达，维持更高比例CD62L^+^低分化表型，具有更长时间的抗肿瘤作用，而随机整合生成的CAR-T细胞在抗原刺激后会迅速分化至效应T细胞，更早出现CAR-T细胞耗竭，丧失肿瘤清除能力[31]。在多项研究中，相较于CAR随机整合的CAR-T细胞，CAR经CRISPR/Cas9定位至PDCD1、CD7或CD38等基因内获得的定向修饰的CAR-T细胞，发生耗竭的细胞比例更低，出现时间更晚，能够在小鼠血液肿瘤模型上更有效地控制肿瘤[32]–[34]。

CRISPR/Cas9使高通量筛选T细胞功能相关基因变得便捷，利用这项技术对细胞进行表观遗传或代谢重编程有助于延缓CAR-T细胞走向终末耗竭。T细胞处于终末分化阶段时转录因子TCF7与LEF1的表达通常被抑制，CRISPR/Cas9敲除CAR-T细胞的DNMT3A能够解除对TCF7与LEF1的甲基化，提高与细胞干性维持相关的转录因子水平，诱导CAR-T呈干细胞样记忆表型，并上调IL-10使线粒体丙酮酸转运载体介导的丙酮酸输入增加，促进CAR-T细胞代谢向氧化磷酸化方式转变，使CD19 CAR-T细胞在抗原长期刺激中维持增殖与肿瘤反应性[35]–[36]。Carnevale等[37]利用CRISPR/Cas9敲除RASA2，促进CAR-T胞内AP-1转录活跃，氧化磷酸化代谢增强并处于效应记忆样分化表型，虽然CAR-T细胞对抗原反应阈值下降但并未快速耗竭，直接调控AP-1组分c-JUN的活性也让CAR-T细胞长期抗肿瘤作用优于对照组[38]。Ye等[39]通过CRISPR激活筛选发现脯氨酸脱氢酶2可重塑CD8^+^ CAR-T细胞脯氨酸与精氨酸代谢，促进线粒体增生从而增加能量储备，让靶向CD22和BCMA的CAR-T细胞在代谢抑制的肿瘤微环境中维持良好的增殖与细胞毒作用。Cox等[40]观察到GM-CSF以剂量依赖性方式增加CAR-T细胞的活化与凋亡，敲除GM-CSF赋予CD19 CAR-T细胞抗凋亡能力与增殖潜力，提高CAR-T体内持久性与清除肿瘤活性。TCR/CD28激活信号由磷脂酶γ1-Ca^2+^-NFATc2a转导通路下传。Trefny等[41]发现敲除SNX9导致NFATc2a核转位减弱，能够降低CD8^+^ CAR-T细胞内NFAT-NR4A1/3-TOX耗竭信号强度，促进其向中枢记忆性细胞分化，并将糖酵解代谢转为脂肪酸和氨基酸氧化，最终增强CD19 CAR-T细胞抗肿瘤作用。一项研究发现转录因子NFAT5可能作为肿瘤微环境高渗感受分子，加剧NFAT1和NFAT2介导CD8^+^T细胞耗竭[42]，利用CRIPSR/Cas9调节T细胞耗竭相关转录因子活性是改善CAR-T耗竭值得尝试的角度。

阻断免疫抑制信号是目前肿瘤免疫治疗思路之一。利用CRISPR/Cas9将CD19 CAR定向整合至PD-1基因治疗r/r B细胞非霍奇金淋巴瘤的临床研究表明，敲除PD-1不仅可以缓解肿瘤PD-L1对CAR-T细胞的抑制，还可以让CAR-T通过与PD-1下调相关的抗肿瘤免疫途径在PD-L1低表达的肿瘤中仍保持优势[34],[43]。敲除与PD-1类似的免疫抑制受体如LAG-3、TIM-3或TIGIT等对T细胞存活与效应功能均有不同程度的提升[44]，但对不同检查点的组合干预还有待进一步研究。肿瘤细胞分泌免疫抑制分子如TGF-β诱导T细胞耗竭，通过CRISPR/Cas9移除CAR-T细胞TGF-β受体Ⅱ可防止CAR-T细胞耗竭，表现出强大的肿瘤杀伤活性[45]。此外，肿瘤微环境低氧引起局部腺苷浓度增加，腺苷通过腺苷A2A受体（A2AR）抑制T细胞免疫应答能力，CRISPR/Cas9敲除A2AR使CAR-T不受缺氧限制，同时增强其JAK-STAT信号通路相关基因表达，提高CAR-T抗肿瘤细胞因子TNF和IFN-γ分泌水平，增强Lewis Y CAR-T细胞体内抗肿瘤能力[46]。

3. 扩大CAR-T应用范围：在血液恶性肿瘤中，B系统肿瘤有CD19、CD20、CD22、BCMA等谱系限制性抗原作为较理想的CAR-T靶点，但T细胞恶性肿瘤与急性髓系白血病（AML）适宜靶点的选择却相当棘手。

在T细胞恶性肿瘤中，CD7和CD5作为靶点能覆盖大部分T系恶性肿瘤，但由于它们在正常T细胞上也表达，针对该类共有靶点的CAR-T存在自相残杀及功能障碍，患者也面临T细胞发育不全致严重免疫缺陷的风险。若能移除正常T细胞上的靶点，则能实现让CAR-T特异性杀伤恶性T细胞的可能。在CAR导入T细胞前使用CRISPR/Cas9敲除CD7可显著改善CD7 CAR-T细胞扩增且不影响其效应。使用HLA配型供者来源的CD7 CAR-T治疗20例儿童和成人r/r T-ALL的Ⅰ期研究显示，中位随访27个月，ORR和MRD阴性CR率分别为95％和85％，7例桥接HSCT，2年无进展生存率和总生存率分别为36.8％和42.3％，6例患者复发（CD7阴性复发4例）；安全方面，观察到5例严重感染和1例4级肠道GVHD[47]。另一项临床研究敲除健康供者来源T细胞的CD7、TCR和HLA-Ⅱ治疗CD7^+^血液肿瘤，11例可评估患者ORR为81.8％，CR/CRi率为64％，3例桥接HSCT，中位随访10.5个月，4例持续CR，3例CD7阳性复发；整个过程中无GVHD、免疫效应细胞相关神经毒性综合征（ICANS）或≥3级细胞因子释放综合征（CRS），有1例输注后93 d因EBV相关弥漫大B细胞淋巴瘤死亡[48]。CD5作为T系肿瘤的另一个热门靶点，虽然表达CD5-CAR的T细胞具有自限性下调CD5以避免自相残杀的特点，但不敲除CD5的CAR-T抗肿瘤能力欠佳。最新一项研究揭示CD5是T细胞效应功能负调控分子，敲除CD5能明显增强CAR-T抗肿瘤效果[49]。应用健康供者来源CD5敲除的CD5 CAR-T细胞治疗r/r T-ALL的Ⅰ期临床数据显示，CR/CRi率为100％（16/16），中位随访14.3个月，4例接受HSCT，3例持续缓解，1例死于感染，而未接受HSCT的12例中2例持续缓解，3例复发，5例和2例分别因感染和血栓性微血管病死亡，存活患者外周血CD5 CAR-T细胞以低于正常T细胞水平持续存在[50]。以上数据提示靶向CD5和CD7的可行性，但需要密切关注患者感染与免疫重建的问题，而桥接移植有助于提升整体疗效。同时我们可以看到治疗T系肿瘤通常采用异基因CAR-T细胞联合CRISPR/Cas9敲除共有抗原及移植排斥相关抗原，因为自体CAR-T应用于该类肿瘤还面临无法获取有效数量的正常T细胞及肿瘤细胞污染CAR-T的问题。不过一些研究团队对自体CD7 CAR-T细胞探索的研究表明，在外周血负荷较低且能够收集到足量T细胞患者中，自体CD7 CAR-T细胞具有与异基因相当的治疗效果且能减低GVHD及感染风险，但对采血时机、CAR-T制备技术以及回输前CAR-T细胞MRD阴性质检等方面有着更为严苛的要求[51]–[52]。

在AML中，CD33、CD123、CLL-1等由于在AML细胞上高表达被认为是具有治疗可能的靶点，但由于AML细胞异质性较高且正常髓系细胞或正常造血干细胞上也有这类抗原的表达，目前CAR-T细胞治疗AML的效果非常有限，并伴随明显的瘤外毒性。近期公布的针对CD33和CD123的CAR-T研究中ORR分别为0～11％[53]–[54]、25％[55]–[56]，获得反应患者后续均出现疾病进展。CLL-1 CAR-T临床反应效果稍好，ORR可达73.3％，但所有患者均出现血液学毒性（30/30），其中29例发生3/4级粒细胞减少症，28例发生3/4级贫血，30例发生血小板减少症，桥接移植可缓解该毒副作用[57]。CD45是一种广泛分布于血细胞高度保守的分化抗原，具有作为泛血液肿瘤靶点的前景。但CD45也在造血干细胞（HSC）上高表达，直接靶向面临严重全血细胞减少，CD45缺失短期对T细胞功能影响较小，但会损害其长期控制肿瘤能力[58]。因此研究人员选择CRISPR碱基编辑技术对T细胞和HSC的CD45表位进行非同义突变，保留CD45生物学功能但确保它们有耐CD45-CAR的能力。CD45 CAR-T细胞联合移植经编辑的HSC在患者来源AML、T系及B系小鼠模型上有出色的肿瘤清除能力与持久性[58]。基于HSCT在AML治疗中的重要作用、CRISPR编辑技术对HSC编辑的可行性，这项研究为CAR-T疗法应用于AML提供了新的设计思路。

三、CRISPR/Cas9技术面临的挑战与解决方向

1. 精准安全的基因编辑：精确控制现有基因编辑技术（表1）的编辑结果依然充满挑战。CRISPR/Cas9脱靶效应指gRNA与非靶向序列局部匹配仍激活Cas9活性，出现计划外的基因编辑[59]，如何实现真正的精准编辑是一直以来的研究热点。

**表1:** CRISPR/Cas9技术与其他基因编辑技术的比较

基因编辑技术	优势	劣势
基于CRISPR/Cas		
CRISPR/Cas9	RNA识别DNA设计与操作简便、编辑效率高、可多重编辑、适合多种细胞类型	脱靶率较高、潜在免疫原性
碱基编辑（nCas9或dCas9）	适合特定单碱基突变、编辑精度高	不便于多重编辑
先导编辑（nCas9）	编辑类型灵活、利用HDR编辑精度高	编辑效率较低、技术较复杂、成本高
CRISPR/Cas12a	可靶向基因范围广、编辑效率高、植物细胞应用广泛	脱靶率较高、潜在免疫原性
锌指核糖核酸酶（ZFN）	蛋白识别DNA编辑特异性较高	操作复杂需设计合成大量蛋白、偏好富含G的靶向序列
转录激活因子样效应因子核酸酶（TALEN）	蛋白识别DNA编辑特异性较高、构建较ZFN简单	操作较复杂、组分大增加递送难度

**注** nCas9：仅有单链切割活性的Cas9蛋白；dCas9：无DNA切割活性，但可结合靶标DNA的Cas9蛋白；HDR：同源定向修复

CRISPR/Cas9技术对特定基因的敲除通常是利用NHEJ途径产生插入缺失突变而成。但这个过程形成的DSB意味诱导染色体易位、缺失或非整倍性的风险，这些事件在CRISPR/Cas9编辑中并不少见[60]。有研究发现原代T细胞编辑后染色体易位发生比例高达总编辑事件1％[61]，染色体缺失发生率可达3.25％[62]。CRISPR衍生技术如碱基编辑器（base editor, BE）和先导编辑基本独立于细胞周期，且几乎不形成DNA双链断裂，不依赖细胞DNA修复机制，具有更低的脱靶率，随着碱基转换和颠换的技术壁垒逐步攻破[63]，新型编辑工具拥有更多的可操控性。不过，最近有证据表明该类编辑器仍存在删除和易位等遗传毒性[64]，但能通过AI辅助蛋白结构预测系统改良编辑器减少不良效应[65]。

工程化改造Cas9蛋白可提升编辑特异性。基于Cas9蛋白晶体结构解析其与靶向及非靶向DNA互作位点特征，对Cas9功能结构域特定氨基酸位点进行定向改造，新型高保真Cas9突变体具有更高DNA靶向特异性[66]。此外，将Cas9与核酸外切酶TREX2偶联，利用外切酶对DNA断裂末端的保护修饰抑制Cas9的重复切割可大幅减少DNA大片段缺失和染色体易位发生[61]。

优化gRNA和人工模板不仅可提升编辑特异性，还有望实现基因编辑在体控制。Longo等[67]开发了一种高通量工具，评估Cas9在人类基因组中大约3 500条gRNA靶向的超过15万个内源性在靶和脱靶位点的切割特征，表明基于目标序列、Cas变体及遗传背景对gRNA进行综合设计是提高编辑结果预测性的有效手段。敲除TREX1基因或使用化学修饰单链DNA模板通过增加HDR比例从而提高编辑的准确性[68]。Lei等[69]采用环状二硫物随机酰化gRNA链上的2′-羟基基团，通过氧化还原操控功能性gRNA释放，实现胞内CRISPR/Cas9系统的开启与关闭控制。

在合适时机引入编辑工具也可降低Cas9相关遗传毒性，Tsuchida等[70]发现在T细胞活化前引入CRISPR/Cas9较活化后引入形成的染色体缺失比例更低，这可能得益于未活化T细胞内高水平p53蛋白存在，可诱导编辑过程发生染色体异常的细胞凋亡。

寻找不同CRISPR系统，拓展基因编辑工具库。FLSHclust识别出全新188种可用于编辑哺乳动物细胞的CRISPR相关系统，展现了CRISPR前所未有的多样性和灵活性[71]。Fanzor是真核生物及相关病毒中类似CRISPR系统的DNA内切酶，可在RNA引导下对人类细胞基因组进行编辑，由于核定位信号强、体积小易递送、且无旁系切割活性的特点，具有实现精准基因编辑的潜力[72]–[73]。

RNA编辑是今后CRISPR/Cas9应用于临床治疗的候选方向。Cas9持续存在的编辑活性使基因组处在较高脱靶风险下，直接作用于半衰期较短的mRNA，在安全性及改变基因表达上具有天然优势[74]。目前对CRISPR系统挖掘的结果也表明自然界存在靶向RNA的蛋白质，进一步开发可用于RNA精准编辑[71]。

2. 安全且有效的递送方式：如何将CRISPR系统高效、准确地递送至目标细胞核内仍是亟待突破的关键问题，目前常单独或组合使用病毒载体、电穿孔或包膜颗粒等方法将gRNA与Cas9引入细胞（表2）。

**表2:** 不同CRISPR/Cas9递送系统的比较

类型	优势	劣势
病毒载体		
慢病毒载体	对分裂及非分裂细胞均有效、载体容量较大	随机整合有潜在遗传毒性、CRISPR/Cas9长期存在于细胞内增加脱靶风险
腺病毒载体	载体容量大、不整合至基因组内安全性高	免疫原性高
腺相关病毒载体	不整合至基因组内安全性高、低免疫原性、具有一定细胞特异靶向性	载体容量小、外源基因表达需要较长时间
非病毒载体		
电穿孔	高效、成本低	细胞机械损伤、无靶向性、无法体内递送
脂质纳米颗粒	低免疫原性、工程化灵活性高	效率、稳定性及组织靶向性待提高
类病毒颗粒	可具备细胞特异靶向性	封装尺寸限制、在体内易被清除、效率待提高
细胞外囊泡	生物相容性高、免疫原性低、稳定性高	工艺复杂、技术要求高、易被肝脏摄取

病毒载体递送CRISPR/Cas9研究时间长，已有不少临床实践经验[75]–[76]。腺相关病毒载体由于不整合至基因组及低免疫原性而具备较高安全性，且不同血清型具不同组织偏好性，但其荷载能力较小；腺病毒载体对外源基因的载量很大，但有较高免疫原性；慢病毒载体也能承载较大的基因片段，可感染分裂与非分裂细胞，但其随机整合至基因组的特点有潜在遗传毒性风险。

非病毒载体是未来递送CRISPR/Cas9的趋势，电穿孔或包膜载体递送CRISPR/Cas9系统在临床中已有初步应用[43],[77]。

不过需注意的是，电穿孔对T细胞的损伤仍是难题，An等[78]研究表明dsDNA在电转中对原代T细胞的细胞毒性由渗透压诱导胞质cGAS-STING通路激活引起，采用改良等渗电转缓冲液可将电转后细胞存活量提升20倍。优化电转条件可为改善非病毒介导的靶向插入提供技术基础，但由于制备条件特性，该技术并不适于体内递送。

随着生物工程技术的发展，包膜递送载体（包括脂质纳米颗粒、类病毒颗粒、胞外囊泡等）的开发如火如荼，其用于体内递送的巨大潜能逐渐显现。目前大多数脂质纳米颗粒（lipid nanoparticle, LNP）仍以体外、局部给药或全身给药靶向肝脏为主[79]，不过最新一项研究表明，优化LNP组分能实现在体靶向肝外组织[80]。把和骨髓细胞亲和力高的分子融入纳米颗粒获得可靶向骨髓的LNP，该载体能将CRISPR/Cas9在镰状细胞病小鼠模型中递送至HSC完成基因编辑，诱导活性血红蛋白生成[80]，目前这项技术在肺[81]和脾脏[82]递送中也取得进展。

类病毒颗粒具备生物膜亲和力与整合风险低的优点。Zhang等[83]将甲流病毒的内含体逃逸肽（HA2）与HIV反式激活转录蛋白（TAT）融合形成辅助蛋白（AP），AP高效促进Cas9进入核内，仅需将编辑系统与细胞共孵育30 min即可完成递送，对原代T细胞及造血祖细胞的基因编辑效率均超过70％，且未观察到细胞转录水平的不良干扰。类病毒颗粒还可用于在体CAR-T细胞生成，将突变型水泡性口炎病毒糖蛋白与靶向T细胞CD3、CD4和CD28的scFv融合形成新型抗体导向包膜载体[84]，静脉注射该载体包装的CAR基因及CRISPR/Cas9系统，能在小鼠体内生成CD19 CAR-T细胞，相较于慢病毒制备的CAR-T细胞，尽管该方式CAR^+^比例较低（约0.5％），但CAR-T细胞克隆均质度更高，可对B细胞进行有效杀伤。

细胞外囊泡是天然纳米级载体，在容量及安全性上具有优势，工程化囊泡是体内递送CRISPR系统研究热点。Liu等[85]利用TAT和血管肽-2双重修饰的细胞外囊泡递送gRNA/Cas9 RNP，成功通过小鼠血脑屏障及血脑肿瘤屏障靶向至胶质母细胞瘤，对其胱甘肽合成酶基敲除效率达到67.2％，显著增强肿瘤对放疗的敏感性。

体内基因编辑蓬勃发展趋势下，递送载体的给药方式、体内代谢动力学、药物相互作用等是今后需要深入研究的方面。

3. 伦理与规范监管：随着CRISPR/Cas9技术在CAR-T细胞中越来越多的应用，伦理和规范监管问题备受关注。由于目前国际上尚无统一的编辑技术管理办法，更依赖各地区各研究单位细致的伦理审查和严格的规范监管来确保技术应用的安全性与合理性。
